# Carotenoid Crystal Formation in Arabidopsis and Carrot Roots Caused by Increased Phytoene Synthase Protein Levels

**DOI:** 10.1371/journal.pone.0006373

**Published:** 2009-07-28

**Authors:** Dirk Maass, Jacobo Arango, Florian Wüst, Peter Beyer, Ralf Welsch

**Affiliations:** Faculty of Biology, Cell Biology, University of Freiburg, Freiburg, Germany; University of Melbourne, Australia

## Abstract

**Background:**

As the first pathway-specific enzyme in carotenoid biosynthesis, phytoene synthase (PSY) is a prime regulatory target. This includes a number of biotechnological approaches that have successfully increased the carotenoid content in agronomically relevant non-green plant tissues through tissue-specific *PSY* overexpression. We investigated the differential effects of constitutive *AtPSY* overexpression in green and non-green cells of transgenic Arabidopsis lines. This revealed striking similarities to the situation found in orange carrot roots with respect to carotenoid amounts and sequestration mechanism.

**Methology/Principal Findings:**

In Arabidopsis seedlings, carotenoid content remained unaffected by increased AtPSY levels although the protein was almost quantitatively imported into plastids, as shown by western blot analyses. In contrast, non-photosynthetic calli and roots overexpressing *AtPSY* accumulated carotenoids 10 and 100-fold above the corresponding wild-type tissues and contained 1800 and 500 µg carotenoids per g dry weight, respectively. This increase coincided with a change of the pattern of accumulated carotenoids, as xanthophylls decreased relative to β-carotene and carotene intermediates accumulated. As shown by polarization microscopy, carotenoids were found deposited in crystals, similar to crystalline-type chromoplasts of non-green tissues present in several other taxa. In fact, orange-colored carrots showed a similar situation with increased PSY protein as well as carotenoid levels and accumulation patterns whereas wild white-rooted carrots were similar to Arabidopsis wild type roots in this respect. Initiation of carotenoid crystal formation by increased PSY protein amounts was further confirmed by overexpressing *crtB*, a bacterial *PSY* gene, in white carrots, resulting in increased carotenoid amounts deposited in crystals.

**Conclusions:**

The sequestration of carotenoids into crystals can be driven by the functional overexpression of one biosynthetic enzyme in non-green plastids not requiring a chromoplast developmental program as this does not exist in Arabidopsis. Thus, *PSY* expression plays a major, rate-limiting role in the transition from white to orange-colored carrots.

## Introduction

Beta-carotene was first isolated from orange carrot roots, which are very high in this carotenoid [1]. The discovery was followed by the elucidation of numerous other carotenoids, and their number still continues to grow [2]. Carotenoids are isoprenoids synthesized by all photosynthetic organisms and some non-photosynthetic bacteria and fungi. In plants, carotenoids are synthesized in plastids, where they are constituents of light-harvesting complexes and photosynthetic reaction centers. They absorb energy from light and thus contribute to the dissipation of excess energy [3]–[5]. Carotenoids are also accumulated in chromoplasts where they act as visual attractants for pollinating insects and seed-distributing animals. In this plastid type, carotenoids are sequestered into sub-organellar structures, which are classified as globular, crystalline, membranous, fibrillar and tubular types according to their architecture (reviewed in [6]).

In many cases the flux of carbon through the carotenoid biosynthetic pathway appears to be controlled by phytoene synthase (PSY), which catalyzes the head-to-head condensation of two geranylgeranyl diphosphate (GGPP) molecules to yield phytoene, the first committed reaction in carotenogenesis. GGPP is a substrate to several other enzymes and can thus enter a number of pathways, leading to the synthesis of tocopherols, chlorophylls, plastoquinones, phylloquinones, and gibberellins.

Carotenogenesis proceeds via phytoene desaturation and cis-trans isomerization to lycopene, catalyzed by two desaturases (phytoene and ζ-carotene desaturase) and one or potentially two isomerases [7]–[9]. Cyclization reactions, mediated by either β- or α-cyclase, introduce β- or ε-ionone end-groups into lycopene, which are then oxygenated (hydroxylated and epoxidated) to form the typical complement of plant xanthophylls (reviewed in [10]).

In chloroplasts, carotenoids and chlorophylls are needed in a defined stoichiometric ratio. The biosynthesis of both pigments, which share GGPP as a common substrate, is thought to occur in a strongly regulated and interdependent fashion. The regulation of PSY occurs at multiple levels: transcription of the *PSY* gene is light-regulated, while the presence of enzymatically inactive PSY within the prolamellar body of etiolated seedlings also indicates posttranslational control [11]–[13].

Most plants seem to contain more than one *PSY* gene, although *Arabidopsis thaliana* only has one. PSY paralogs can play specialized roles in chromoplast development, as found in developing tomato fruits [14]–[17]. Functional diversification of PSY was also found in rice and maize, where one of three PSYs is specifically involved in stress-induced ABA formation [18], [19]. Furthermore, two *PSY* genes are found in carrots (*Daucus carota*; [20]), cassava (unpublished), and are also found annotated for poplar and grapevine.

Because of PSY's key role at the entry point into carotenogenesis, it is not surprising that biotechnological approaches to increase carotenoid levels aim at increasing its expression. However, overexpression of *PSY* can affect photosynthetically active and non-active tissues in different ways. Tomato plants overexpressing tomato *PSY1* constitutively in all tissues, for instance, showed only slightly increased leaf carotenoid levels [21], while accumulation of total carotenoids during fruit ripening was much more pronounced [22]. Most recent transformation experiments to increase total carotenoid content in non-green tissues used tissue-specific promoters to circumvent possible adverse affects, such as a dwarf phenotype, as has been observed in some transgenic lines when using the constitutive, non-tissue-specific *CaMV35S* promoter [21]. Tissue-specific promoters were used to raise the carotenoid content in Arabidopsis seeds [23], canola seeds [24], potato tubers [25], and rice endosperm [26]. In the latter the choice of the *PSY* gene was crucial to attain high carotenoid levels [27], while increased expression of the phytoene desaturase *CrtI* was not effective [28]. However, PSY may not be rate-limiting in all tissues, as exemplified in transgenic potato tubers, where overexpressed lycopene β-cyclase had a dominating effect [25].

In the present work, we set out to systematically investigate the impact of PSY on carotenoid formation in green and non-green tissues, using transgenic *A. thaliana* seedlings, roots and calli, as well as transgenic *D. carota* storage roots. Analysis of RNA and protein expression, combined with quantitative carotenoid analysis, revealed profound differences in the responsiveness of carotenogenesis to increased PSY protein levels. PSY turned out to be rate-limiting in non-green tissues but not in green tissues. We propose that this difference is due to the capability of non-green plastids but not chloroplasts to form carotenoid crystals. In non-green plastids crystal formation is simply driven by enhanced carotenoid biosynthesis, and does not require the activation of intrinsic developmental programs to drive chromoplast development.

## Results

### PSY expression is rate-limiting in calli but not in green seedlings

**Figure 1:**
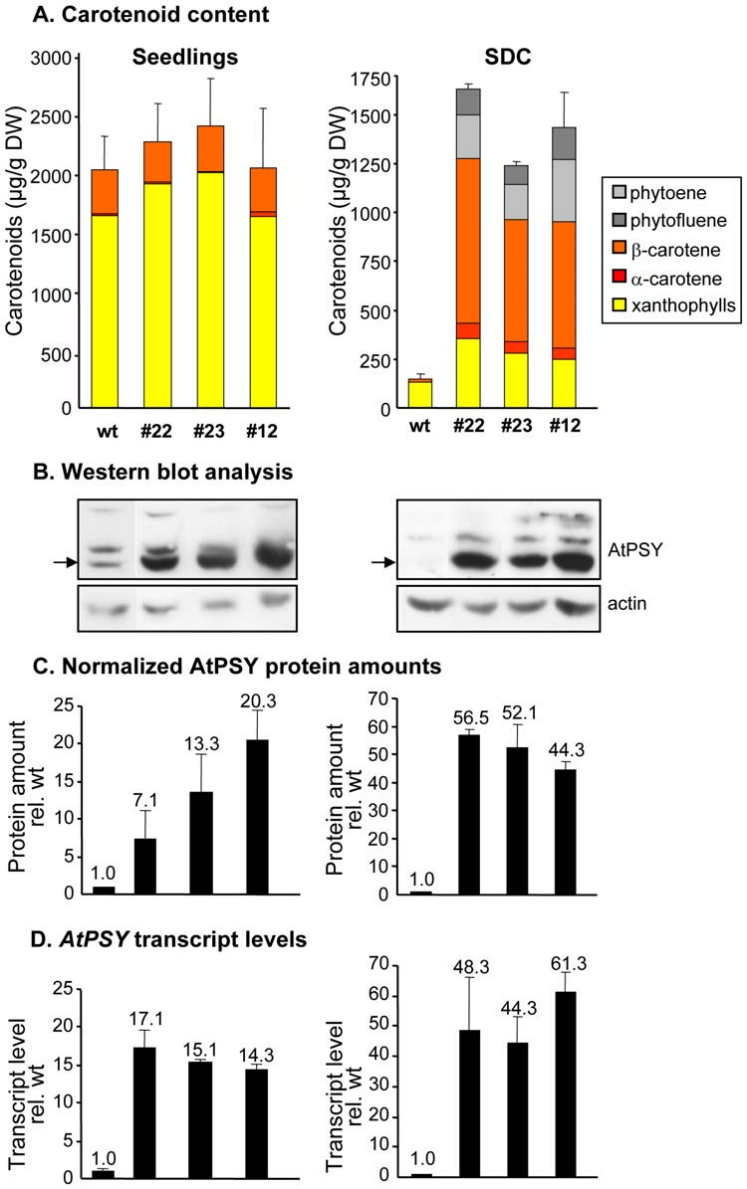
Carotenoid content, AtPSY transcript and protein levels in *AtPSY* overexpressing lines. Seedlings (left side) from wild type (wt) and from three *35S::AtPSY* lines (#12, #22, #23) were grown for two weeks on MS plates. For seed-derived calli (SDC), seedlings were germinated on callus-inducing medium under long-day conditions for 5 days, followed by 16 days of darkness. A: Carotenoid content as determined by quantitative HPLC. B: Western blot analysis of AtPSY protein levels using 60 µg of protein extracts. The signal corresponding to the imported AtPSY protein is marked with an arrow. Protein levels of actin are shown as a loading control. C: Internal normalization of AtPSY relative to actin in the corresponding sample. D: *AtPSY* transcript levels determined by Real-Time RT-PCR using total RNA. Transcript levels were normalized to 18S rRNA levels of the corresponding sample and expressed relative to the content in the wild type. Data represent the mean of three biological replicates.

We investigated the effects of *PSY* overexpression in green and non-green tissues, seedlings and calli of Arabidopsis lines expressing the *PSY* ORF from Arabidopsis *(AtPSY)* under the control of the constitutive, non-tissue-specific *CaMV35S* promoter *(35S::AtPSY)*. Seedlings of three *35S::AtPSY* lines grown for two weeks were phenotypically indistinguishable from wild-type seedlings. Carotenoids as well as *AtPSY* transcript levels were determined by HPLC and Real-Time RT-PCR, respectively. Although *AtPSY* transcripts increased 14 to 17-fold, carotenoid levels and accumulation patterns as well as chlorophylls remained unchanged as compared to wild-type seedlings (Figure 1A and D, left).

**Figure 2:**
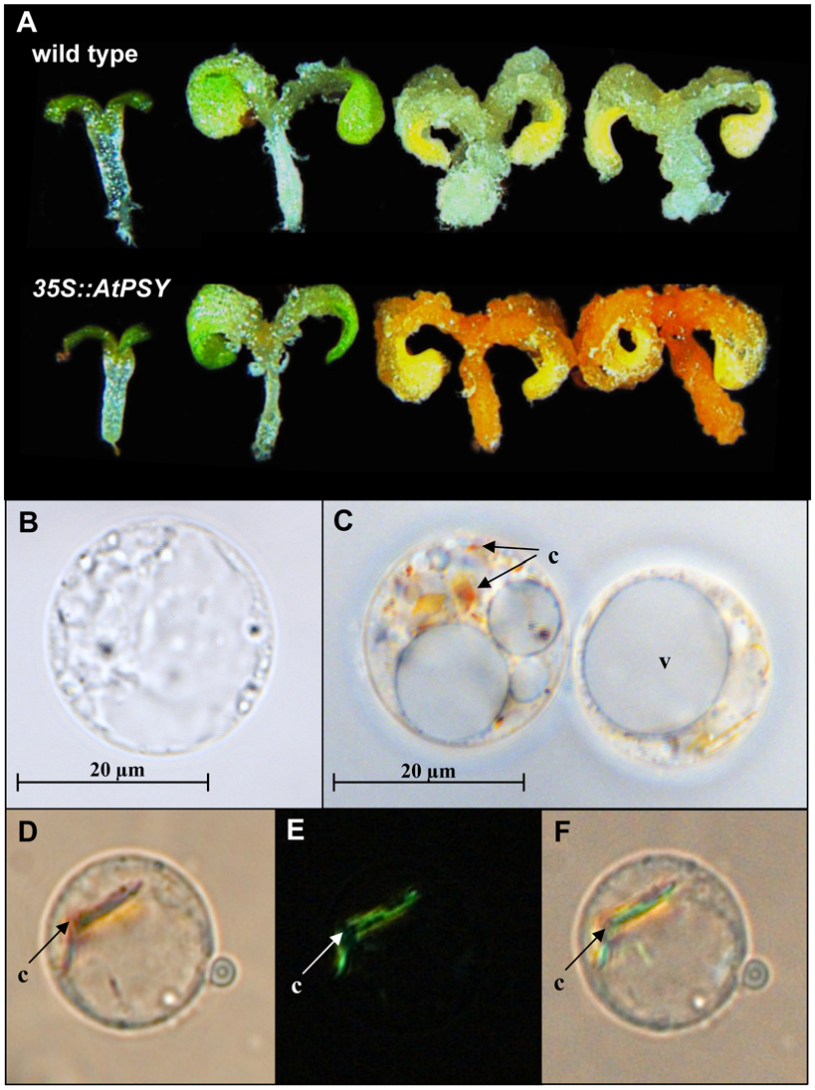
Development of carotenoid crystals during SDC development. A: Development of SDC from wild type and one *35::AtPSY* line. Seedlings after 5 days development in light (left) and after 7, 12 and 16 days etiolation are shown. C–F: β-carotene crystal formation in SDC generated from the *35S::AtPSY* line after 16 days in darkness. In addition to small bead-like, orange colored structures (C), the formation of large bar-shaped structures was observed in SDC protoplasts by light microscopy (D). As confirmed by polarization microscopy, these structures represent carotenoid crystals (E); an overlay of D and E is shown in F. A protoplast from wild-type SDC is shown in B. c, carotenoid crystals; v, vacuole.

Calli were induced from germinating seeds, leaves, stems and roots of *35S::AtPSY* lines as well as from the wild type. Since all the transgenic calli responded almost equally with respect to carotenoid accumulation (data not shown), seed-derived calli (SDC) were chosen for all further experimentation. These calli were generated by germinating seeds on callus-inducing medium for 5 days under long-day conditions, followed by 16 days in darkness. During etiolation, the transgenic lines developed intensely orange-colored calli, while those of the wild type were much less colored (Figure 2A). Quantitative HPLC revealed an approximately 10-fold increase in carotenoid levels compared to the wild type (Figure 1A, right), together with changes in carotenoid accumulation patterns. While wild-type SDC accumulated mainly xanthophylls and only trace amounts of β-carotene, the latter was the main constituent in *35S::AtPSY* SDC, followed by xanthophylls and carotene intermediates, like phytoene and phytofluene.

### PSY protein levels correlate with increased carotenoid (β-carotene) levels in seed-derived calli but not in leaves

We compared the response of leaves and SDC to *AtPSY* overexpression by determining the AtPSY protein levels in both tissues using affinity-purified anti-AtPSY antibodies.

**Figure 3:**
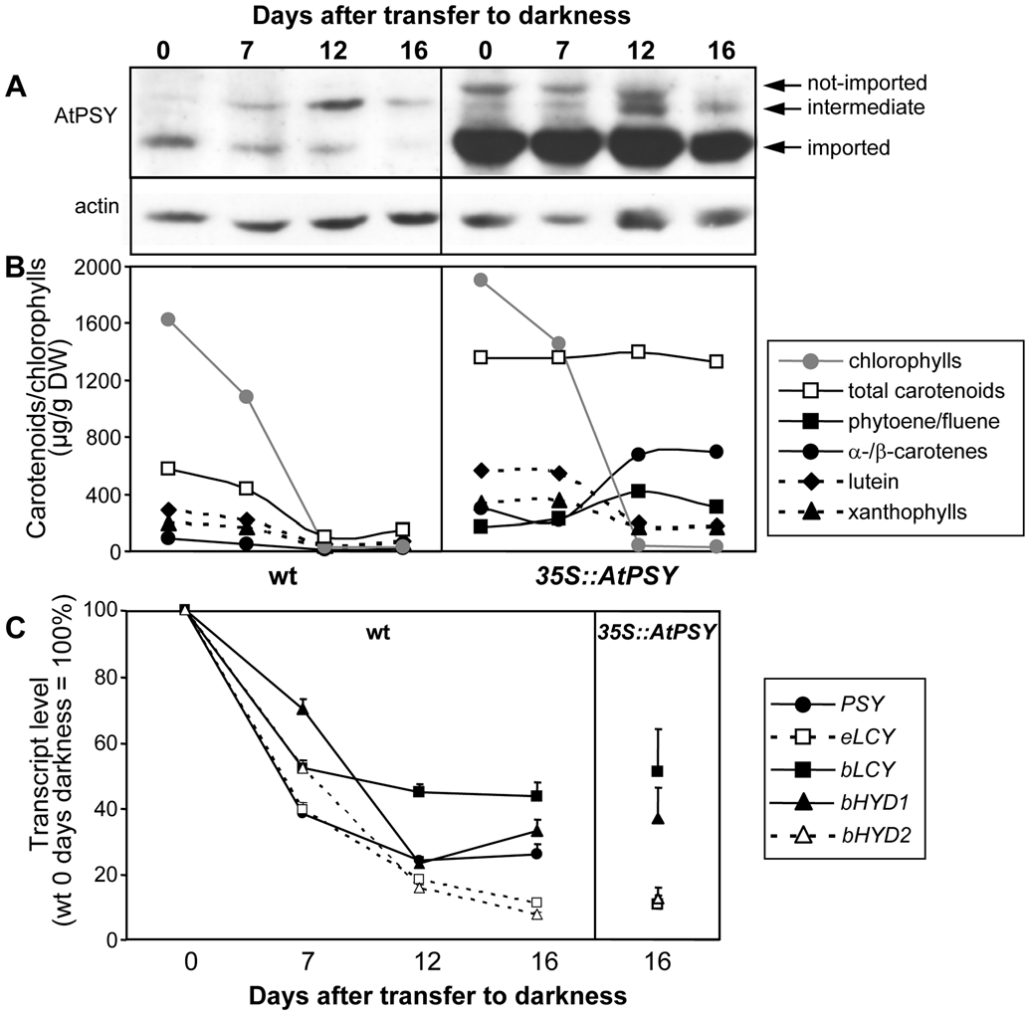
Carotenoid and chlorophyll content, PSY protein and transcript levels during SDC development. Seeds from wild type (left) and a *35S::AtPSY* line (right) were germinated on callus-inducing medium under long-day conditions for 5 days, followed by 16 days of darkness. Samples were taken at the time of transfer to darkness (0 days) and after 7, 12 and 16 days etiolation. A: Western blot analysis of AtPSY protein levels using 60 µg of SDC protein extracts. The protein levels of actin are shown as a loading control. B: Carotenoid and chlorophyll content in SDC as determined by quantitative HPLC. C: Real-Time RT-PCR using total RNA isolated from wild type and *35S::AtPSY* SDC. Transcript levels were normalized to 18S rRNA levels of the corresponding sample and expressed relative to the content in the wild type after 5 days of growth in the light. Expression level for *AtPSY* in *35S::AtPSY* is 20-fold compared to the wild type after 5 days in the light (not shown). Data represent the mean of three technical replicates. *PSY*, phytoene synthase; *eLCY*, lycopene ε-cyclase; *bLCY*, lycopene β-cyclase; *bHYD1/2*, βcarotene hydroxylase 1/2.

Three AtPSY bands were observed on western blots with seedlings as well as with SDC (Figure 1B and 3A). The 42 kD band corresponds to the mature protein after import into plastids. It co-migrates with the radiolabelled in-vitro translation product lacking the transit peptide on PAGE gels [29]. Similar internal standards also allowed relating the uppermost band (48 kD) to the unprocessed cytoplasmatic protein (data not shown). An intermediate form (ca. 45 kD) was observed in most cases but was not further investigated.

Both tissue types imported the overexpressed protein almost quantitatively into plastids; the concentration of unprocessed precursors did not rise. Therefore, the translocation step is not rate limiting and is thus not the cause for the differential response.

In fact, both transcript and protein levels were found to be substantially higher in green leaves of the transgenic lines as compared to the wild type (Figure 1B). This means that the high carotenoid content in seedlings prevents the overexpressed AtPSY from raising carotenoid levels further. In the wild type, even low levels of AtPSY ‘saturate’ the system. This contrasts with the situation in calli, where *AtPSY* overexpression is capable of very substantially increasing the low carotenoid levels present in the wild type. Therefore, in green tissues, a relatively small amount of AtPSY is sufficient to produce high carotenoid levels while in SDC a much higher amount of AtPSY is needed to drive carotenoid levels up to seedling levels. *AtPSY* expression is thus rate-limiting in photosynthetically inactive calli but not in green seedlings.

### Rerouting of the carotenoid biosynthetic pathway in 35S::AtPSY SDC leads to β-carotene accumulation

We investigated carotenoid and transcript levels of carotenoid pathway genes during the process of SDC formation from wild-type plants and siblings of one *35S::AtPSY* line (Figure 3B, C). Data were collected after growth in the light and during subsequent etiolation for 7, 12 and 16 days. This revealed that during the light period, SDC of the *35S::AtPSY* line had accumulated more than double as much carotenoids as wild-type SDC. Although β-carotene content increased about 3-fold compared with wild-type SDC at this point, it represented only 20% of total carotenoids, while xanthophylls contributed about 66%. Phytoene and phytofluene, which were absent in wild-type calli, were already present at this stage. During the subsequent 16 days of growth in the dark, xanthophylls decreased to about 25% of total carotenoid content, while β-carotene and to a minor extent also α-carotene, increased strongly, contributing to ca. 50% (or 650 µg/g dry weight) of total carotenoid content after 16 days. Remarkably, this pattern shift did not affect total carotenoid content, which remained as high as before.

In contrast, under the same regime, total carotenoids in wild-type SDC decreased by about 75% due to losses across all carotenoids determined. With the disappearance of chlorophylls (Figure 3B) carotenoids became visible (Figure 2A).

Hydroxylation of β-carotene is strongly downregulated under dark conditions in both wild type and transgenics, as revealed by Real-Time RT-PCR (Figure 3C). Similarly, the transcripts of ε-cyclase fell to about 10% of the light levels, while β-cyclase levels were less affected (ca. 50%), and phytoene desaturase, ζ-carotene desaturase and carotenoid isomerase decreased to about 30% (data not shown). In *35S::AtPSY* SDC, expression of the transgene was 20 times higher than in the wild type, while transcript levels of the other carotenoid pathway genes decreased to the same low levels as wild-type SDC (Figure 3C, right). This indicates that the strong β-carotene accumulation results from *AtPSY* overexpression accompanied by rerouting of the pathway towards the β-branch. The shift is caused by expression changes occurring during the transition to non-green callus cells and not by changes induced by *AtPSY* overexpression.

The decrease of carotenoid levels reflects carotenoid turnover. One interpretation is that decreasing levels of AtPSY and other carotenoid biosynthetic enzymes in wild-type SDC can no longer compensate for carotenoid turnover unless the rate of biosynthesis is increased by *AtPSY* overexpression.

### In SDC expressing AtPSY constitutively β-carotene is deposited in crystals

In chromoplasts, carotenoids are deposited in specialized structures such as plastoglobules, fibrils, crystals or proliferated membranes. Since Arabidopsis cannot form chromoplasts, we set out to determine the mode of carotenoid deposition in plastids of dark-grown *35S::AtPSY* SDC accumulating ca. 1800 µg/g dry weight of these compounds, which is well in the range of crystal formation in chromoplasts. Carrots, for instance, contain 2000 to 4000 µg carotenoids/g dry weight [30] and tomatoes accumulate ca. 2000 µg/g dry weight [22].

Microscopy revealed that wild-type protoplasts contained unpigmented proplastids (Figure 2B) while orange-colored particles were present in small numbers (1–5 per cell) in *35S::AtPSY* protoplasts (Figure 2C). As shown in Figure 2 (D–F), polarization microscopy revealed that the carotenoid particles in *35S::AtPSY* SDC were birefringent, which is distinctive of their crystal nature.

### Carotenoid crystal formation in roots

**Figure 4:**
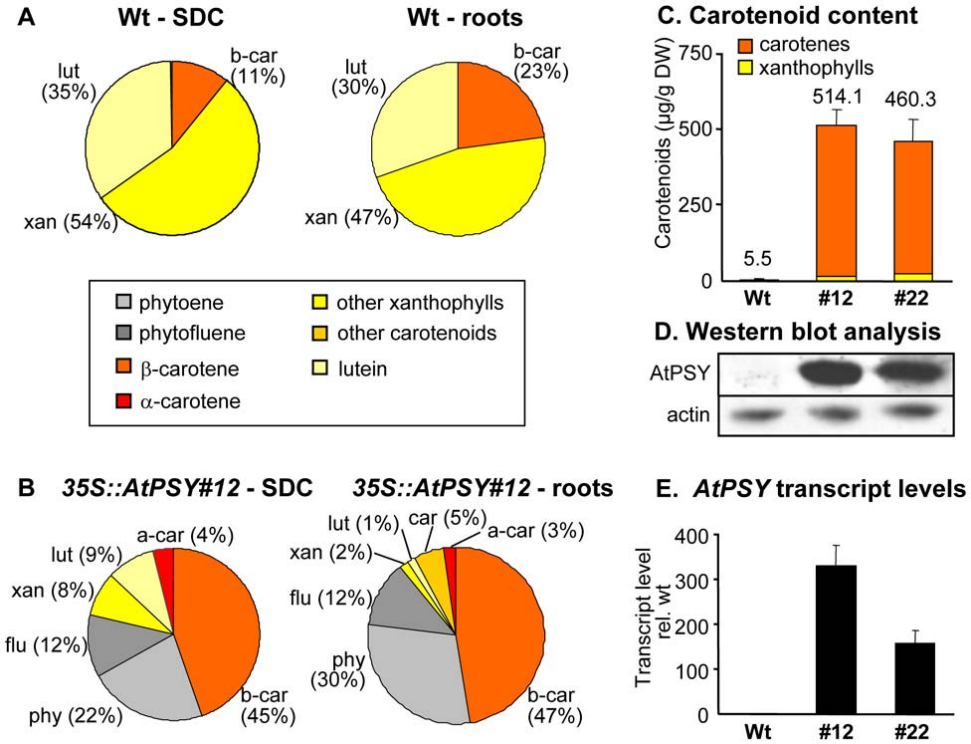
Carotenoid accumulation in roots of *35S::AtPSY* lines. Arabidopsis wild type (wt) and two *35S::AtPSY* lines (#12, #22) were grown hydroponically for two weeks; roots were harvested and used for analysis. Root carotenoid amounts (C) and a comparison of carotenoid composition patterns in SDC and roots from wild type (A) and *35S::AtPSY* line#12 (B) are shown. Percentages of single carotenoids or carotenoids groups relative to total carotenoid amount are given in brackets. Phy, phytoene; flu, phytofluene; β-car, β-carotene; α-car, α-carotene; xan, other xanthophylls; car, other carotenoids; lut, lutein. D: Western blot analysis of AtPSY protein levels using 60 µg of root protein extracts and anti-AtPSY antibodies. The protein levels of actin are shown as a loading control. E: *AtPSY* transcript levels were normalized to 18S rRNA levels of the corresponding sample and expressed relative to the amounts detected in wild-type roots. Data represent the mean of three biological replicates.

Given that SDC reflect the situation in non-green plant cells, roots should respond similarly to *PSY* overexpression. In fact, as shown in Figure 4C, wild-type roots accumulated only trace amounts of carotenoids while those of *35S::AtPSY* lines accumulated ca. 100 times more. In addition, the carotenoid composition pattern was very similar to SDC, consisting mainly of β-carotene and the intermediates phytoene and phytofluene (Figure 4A). Just like in callus tissues, carotenoid increase in roots correlated with increased AtPSY protein, which was almost undetectable in wild-type roots (Figure 4D). Real-Time RT-PCR showed that carotenoid accumulation correlated with increased expression levels of *AtPSY* (Figure 4E) but did not affect expression levels of other carotenoid genes (*AtPDS, AtZDS, AtbLCY, AteLCY, AtbHYD1, AtbHYD2*; Figure S1). Polarization microscopy of protoplasts prepared from roots of *35S::AtPSY* lines revealed an identical mode of carotenoid sequestration into birefringent carotenoid particles (Figure S2). Thus, roots mirror the situation in SDC almost perfectly.

### Carotenoid composition patterns and PSY expression in roots of cultivated carrots and Arabidopsis *35S::AtPSY* lines are similar

The storage root of orange carrots *(Daucus carota)* represents the most popular β-carotene accumulating tissue. The molecular basis of the massive carotenoid accumulation in carrots is still unclear. Path analysis on the offspring of crosses between white and orange-rooted cultivars suggested that in white roots the capability for phytoene formation was the limiting factor in carotenoid accumulation [31].

**Figure 5:**
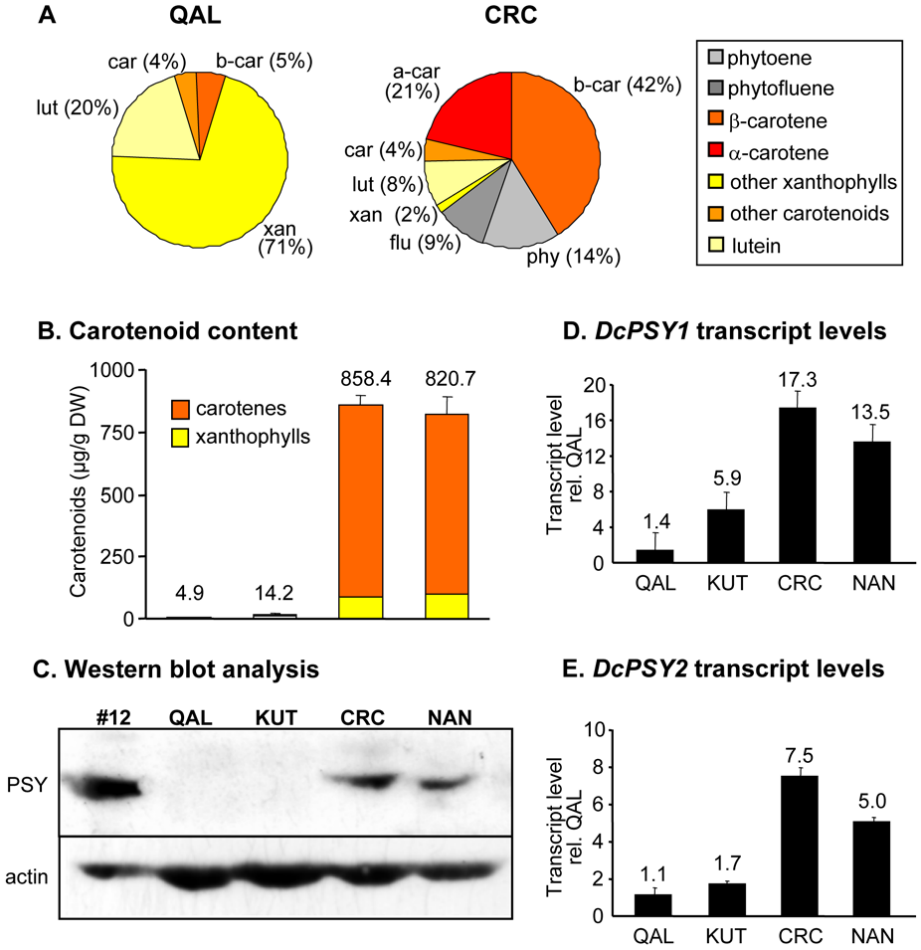
Carotenoid accumulation in white and orange-colored carrot roots. Two carrot varieties with white colored roots (QAL, Queen Anne's Lace, KUT, Küttiger) and two varieties with orange-colored roots (CRC, Chatenay Red Cored, NAN, Nantaise 2) were grown for 9 weeks. Roots were harvested and used for the quantification of carotenoids by HPLC (B). Carotenoid composition patterns for QAL and CRC roots are shown in A; the pattern for KUT was similar to QAL while that for NAN was similar to CRC. C Western blot analysis using 60 µg of root protein extracts from carrots and one *35S::AtPSY* line (#12). Affinity-purified anti-AtPSY antibodies were used. Actin protein levels are shown as loading control. Expression levels of *DcPSY1* (D) and *DcPSY2* (E) in carrot roots as determined by Real-Time RT-PCR. Transcript levels were normalized to 18S rRNA levels of the corresponding samples and are expressed relative to the expression level determined in roots of QAL. All data represent the mean of three biological replicates.

To test the hypothesis we analyzed a white-rooted wild carrot (*D. carota subsp. carota*, Queen Anne's Lace, QAL) and a cultivated variety with white roots (*D. carota subsp. sativus* var. Weisse Küttiger, KUT), as well as two orange-rooted cultivars (*D. carota subsp. sativus*, var. Chatenay Red Cored, CRC, and Nantaise 2, NAN). As shown in Figure 5 (A, B), white-rooted carrots and Arabidopsis wild-type roots were similarly low in carotenoids (6 µg/g DW for Arabidopsis wild type, 5 for QAL, and 14 for KUT), which consisted mostly of xanthophylls and only a minor proportion of β-carotene. Just like in Arabidopsis roots, carotenoid intermediates and α-carotene were virtually absent. The carotenoid levels in cultivated carrot roots (CRC, NAN) of 9-week old plants were as high as in SDC or roots overexpressing *AtPSY* (Figure 5B; compare Figure 4C and 1A). They accumulated around 800 µg/g total carotenoids with about 42% β-carotene, and were characterized by the presence of the pathway intermediates phytoene and phytofluene. The only difference was α-carotene, which accounted for about 20% of total carotenoids in carrots but only 5% in roots of Arabidopsis *35S::AtPSY* lines.

The western blot analyses again mirrored the situation met in roots of Arabidopsis *35S::AtPSY* lines. While in white-rooted carrots PSY was below the detection limit, roots of the orange-rooted varieties showed a strong PSY signal (Figure 5C). We cloned the *PSY* cDNAs from QAL and CRC to make sure that antibody specificity and enzymatic activity were not affecting the interpretation of the results. Two *PSY* genes are present in the carrot genome (*DcPSY1, DcPSY2*; [20]), and they were identical in QAL and CRC.

We investigated whether the increase of PSY protein correlated with *PSY* transcript levels by Real-Time RT-PCR (Figure 5D, E). While transcript levels of *DcPSY2* were almost equivalent in the white-rooted carrots QAL and KUT, *DcPSY1* transcript levels were about three times higher in KUT than in QAL. In the orange-rooted cultivars CRC and NAN, *DcPSY1* transcript levels were 17 and 14-fold higher than in QAL roots, while *DcPSY2* transcript levels were 8 and 5-fold higher, respectively.

### Root-specific *PSY* expression in white carrots leads to yellow-colored roots

Seemingly, the correlation of PSY protein abundance and carotenoid levels in non-photosynthetic tissues of Arabidopsis and carrot roots represents a common theme. Differential expression of other carotenogenic genes does not seem to be required to drive carotenoid overproduction, as can be concluded from comparative transcriptional analysis conducted with several carrot cultivars differing in root color [32].

**Figure 6:**
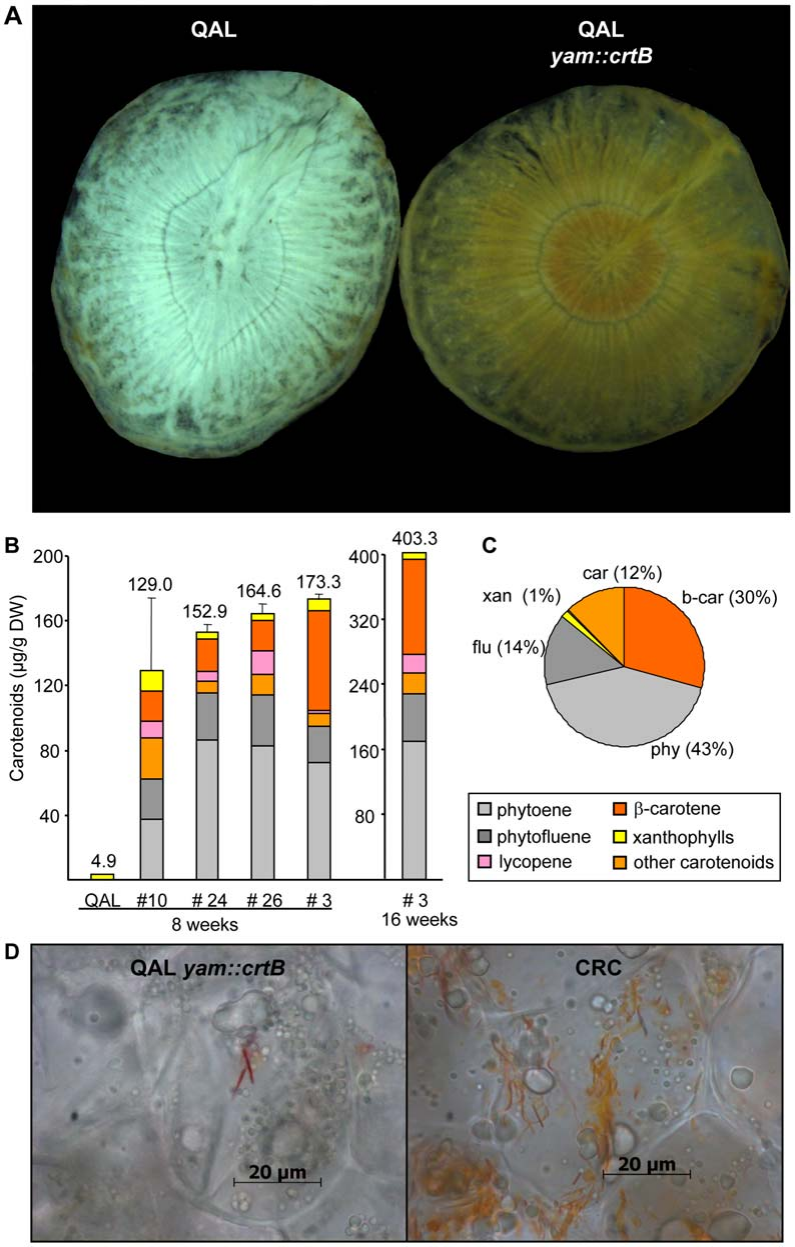
Root-specific overexpression of *PSY* in QAL. White-rooted wild carrots (QAL, Queen Anne's Lace) were transformed with a construct containing the bacterial PSY *crtB* downstream of a root-specific promoter from yam. A: Transverse section of roots from untransformed QAL (left) and *yam::crtB* line #3 (right) from 16-week old plants. B: Carotenoid content and composition pattern of untransformed QAL and four different *yam::crtB* QAL lines as determined by HPLC. The age of the plants is indicated below the diagram. The values represent the mean of two biological replicates. C: Relative percentage of carotenoids in 16-week-old plant of *yam::crtB* line #3. For abbreviations, see Figure 4. D: Light microscopy of root thin section of a *yam::crtB* line (left) and CRC (right).

Consequently, increased PSY in a white-rooted carrot background should produce colored carrot roots. To prove the point we generated transgenic QAL lines overexpressing the bacterial PSY *crtB* under the control of a storage root-specific promoter cloned from yam (*Dioscorea spec*.; Arango et al., unpublished; *yam::crtB*). In fact, roots of about 8-week old heterozygous T1 transformants exhibited intense yellow color (see Figure 6A). However, the carotenoid composition pattern showed some differences compared to orange carrot roots. β-carotene accounted for only 10% of total carotenoids in most lines, with only one line *(yam::crtB#3)* containing about 30% β-carotene (Figure 6B, C). Carotene intermediates, mainly phytoene, but also phytofluene, ζ-carotene, and lycopene predominated in roots of all lines, and α-carotene was absent. The pattern remained constant during prolonged growth while carotenoid levels increased from about 180 µg/g DW at 8 weeks to 400 µg/g DW at 16 weeks.

Even though carotenoid content was lower in roots of *yam::crtB* lines than in orange carrot roots, carotenoid sequestration into crystals followed the same pattern as in roots of cultivated carrots (Figure 6D). From this we conclude that only increased PSY protein levels are required to attain both high carotenoid levels as well as crystal formation, while carotenoid composition patterns may vary.

## Discussion

### AtPSY expression in green and non-green tissues: Effects on carotenoid formation

The expression of *AtPSY* results –as we show– in a differential response in green and non-green plant cells with respect to carotenoid formation. While seedlings were essentially unaffected, carotenoid content in non-green SDC and roots increased by 10-fold and ca. 100-fold over wild-type levels, respectively (Figure 1, 4). In absolute terms, chloroplasts can synthesize very large amounts of carotenoids (2000 µg/g) at low levels of *AtPSY* expression. Non-green plastids accumulate carotenoids less effectively, requiring strongly elevated AtPSY levels to reach levels of 500 (roots) to 1800 (SDC) µg/g dry weight.

This differential response is not due to limitations in the import machinery, since AtPSY pre-proteins did not accumulate in any of the tissues examined. This finding points towards fundamental differences in carotenoid storage mechanisms. In chloroplasts, carotenoid is generally found in protein-bound form, mainly in light-harvesting complexes accommodating carotenoids and chlorophylls [33], [34]. Protein binding of carotenoids may impact on biosynthetic capacity directly or indirectly and provide a sequestering site for carotenoids, allowing highest levels of accumulation in plastid membranes without interfering with their physicochemical properties. Sequestration into light-harvesting complexes most probably also affects carotenoid biosynthesis by shifting the balance of pathway products while at the same time protecting the bound carotenoids from being catabolized. Moreover, PSY is only active when bound to membranes, as shown for daffodil chromoplasts [29], [35]. Accordingly, PSY was essentially inactive in prolamellar bodies of etioplasts while its enzymatic activity was elicited upon the formation of thylakoid membranes [11].

Carotenoid catabolism, which is initiated by carotenoid oxygenases, can influence carotenoid accumulation profoundly [36]–[38], and may be comparatively higher in non-green cells. Here, increased PSY levels or enzymatic activity might be able to override carotenoid turnover, leading to elevated steady-state carotenoid concentrations. It is conceivable that carotenoid stability may be very significantly increased when carotenoids accumulate in suborganellar structures, such as fibrils [39] or crystals, in the absence of functional carotenoid-binding photosynthetic proteins.

### Increased PSY levels lead to carotenoid crystal formation in non-green cells

Carotenoid crystals form in SDC and roots from *AtPSY* overexpressing lines. During the formation of SDC, crystal buildup is supported by reduced carotenoid diversity through the concerted downregulation of β-hydroxylases and ε-cyclase expression, and to a much lesser extent β-cyclase (Figure 3). This process favors β-carotene formation until enough is enriched at the site of formation, causing the β-carotene to leave the lipophilic phase of the membrane and form crystals. Roots of transgenic lines behaved very similarly. In both cases, carotenoid accumulation is due to increased levels of the rate-limiting enzyme PSY although without impacting on the expression of other carotenoid genes, which remained unchanged in SDC and roots of *35S::AtPSY* lines relative to the corresponding wild-type tissues.

A similar mode of carotenoid deposition occurs during the conversion of chloroplasts to chromoplasts in tomato fruits, for instance, which is accompanied by the fruit-specific upregulation of tomato *PSY1*. Here, due to concurrent downregulation of ε and β-cyclases [15], [40], lycopene accumulates in crystalline bodies within the chromoplast [41], [42]; they possibly show up as small, bead-like structures at an early stage of formation [43]. Similar structures were also observed in SDC and root protoplasts of *AtPSY* overexpressing lines (Figure 2, Figure S2). The formation of β-carotene crystals in these cells seems to be solely driven by the overexpression of *AtPSY*, not requiring additional components of a chloroplast or chromoplast developmental program that does not exist in Arabidopsis.

### Similarities between AtPSY overexpressing tissues and the cauliflower OR mutant

The accumulation of large amounts of β-carotene in the form of crystals is strikingly similar to the cauliflower *OR* mutant [44]. However, the *OR* gene is not biosynthetic but encodes a plastid-localized protein containing a DnaJ Cys-rich domain, making the OR protein a likely constituent of the plastidic chaperone system [45]. Indicative for the functionality of the underlying principle, potatoes transformed with the mutated *OR* gene showed increased β-carotene accumulation in tubers [46]. Also, crystal formation is maintained in cauliflower *OR* calli [47], [48].

It is interesting to note that a very similar effect, including altered plastid morphology, can be achieved in non-green Arabidopsis cells by increasing the carotenoid biosynthetic capacity. Carotenoid biosynthetic genes are not upregulated in the *OR* mutant [44], and the phytoene biosynthetic capacity is not increased, as shown using the phytoene desaturase inhibitor norflurazone [47]. In both cases β-carotene crystal formation is the common principle, although the underlying mechanisms may be different. Yet, the resulting effect, namely deposition of β-carotene in a form that is less accessible to catabolic reactions, might be the same. Alternatively, sequestration of β-carotene into crystals may shift local chemical equilibria, thus enhancing the flux of carbon through the pathway. Targeted metabolic profiling of carotenoid degradation products should allow distinguishing between these possibilities.

### Why are carrots orange?

While tomato chromoplasts develop from chloroplasts during fruit maturation, chromoplasts of carrot roots emerge from proplastids with low carotenoid content [49]. Despite these differences in origin, in both cases carotenoids are sequestered as crystals [50], [51].

As we were able to show, *PSY* overexpression can lead to carotenoid accumulation in Arabidopsis roots, matching orange carrots almost perfectly, both in qualitative and quantitative terms. The presence of high PSY levels in orange carrot roots, while undetectable in white-rooted cultivars, provides further support for its proposed key role. Furthermore –again similar to Arabidopsis roots– increased PSY seems sufficient to explain heightened carotenoid accumulation, since transcript levels of other carotenogenic genes do not increase in orange as opposed to white-rooted carrots [32]. This is in agreement with the conclusions drawn from pathway analyses performed by Santos et al. suggesting that the primary difference between white and orange carrots resides in an enzymatic change blocking carotenoid accumulation early in the pathway [31]. Our results suggest that in white-rooted carrots low PSY maintains carotenoid biosynthesis at a low level while in orange carrots high PSY is responsible for the very large amount of accumulating carotenoids.

To confirm the hypothesis, white carrots were transformed to overexpress *PSY* (*CrtB*) in the roots. As expected, their roots showed strongly increased carotenoid levels. However, in contrast to Arabidopsis roots and SDC of *35S::AtPSY* lines the carotenoid composition pattern was characterized by a higher proportion of carotene intermediates like phytoene, phytofluene and ζ-carotene, pointing towards rate-limiting carotene desaturation. Similarly, lycopene which was absent in orange-rooted cultivars and lower levels of β-carotene accumulation indicated lower cyclization capacity. Furthermore, the high proportion of α-carotene in orange-rooted cultivars was neither found in roots of the transgenic carrots nor in non-green tissues of *AtPSY* overexpressing Arabidopsis lines. However, such pathway differences are known in carrot germplasm [52]. Hence, we propose that PSY protein overexpression is the main driving force behind the orange phenotype. Orange carrots are believed to have been domesticated from white cultivars in the 16^th^ century [53], and it seems plausible that the orange phenotype we all know originated from one single mutation event.

## Materials and Methods

### Arabidopsis transformation and callus generation

The vector *pCAMBIA1390-35S* was obtained by subcloning the *CaMV35S* promoter from the vector mAV (kindly provided by Marta Rodriguez Franco, University of Freiburg, Germany) into *pCAMBIA1390*. For *pC1390-35::AtPSY*, the *AtPSY* cDNA (accession no L25812; [54]) was subcloned into *pCAMBIA1390-35S*. *Arabidopsis thaliana* ecotype Wassilewskija was transformed by vacuum infiltration [55]. Homozygous T2 progenies were identified by the segregation pattern of the corresponding T3 progenies on hygromycin (30 µg/ml) containing MS plates.

SDC were generated essentially as described [56]. Ten milligrams of surface-sterilized seeds were plated onto petri dishes (145 mm diameter) containing SDC medium (4.33 g/L MS basal salts/KOH, pH 5.8, 3% [w/v] sucrose, 0.1% [v/v] Gamborg B5 vitamins, 0.5 mg/L 2,4-D, 2 mg/L indole-3-acetic acid, 0.5 mg/L 2-isopentenyladenine, 0.4% [w/v] phytagel). Seeds were germinated under long-day conditions (16 h light/8 h dark, 26°C) for 5 days and incubated for 16 days in darkness.

Seedlings were obtained by plating 7.5 mg surface-sterilized seeds onto MS medium. Petri plates were sealed and the seedlings grown under long-day conditions for 14 days. Seedlings, SDC and roots were ground in liquid nitrogen immediately after harvest and stored at −70°C for further analysis.

### Plant material and growth

Carrot seeds were obtained from the following sources: Queen Anne's Lace, Richters Herbs (Goodwood, Canada); Küttiger, Dreschflegel Saatgut (Witzenhausen, Germany), Chatenay Red Cored, B&T World Seeds (Aigues-Vives, France), Nantaise 2, Freya (Solingen, Germany). Carrots were grown in soil under long-day conditions. Roots of 9-week old carrot plants were removed from the soil, ground in liquid nitrogen and stored at −70°C for further analysis.

### Carrot transformation

Transgenic QAL lines were transformed with *pCAMBIA1305.2-yam::crtB* as described [57]. Transformation was carried out using pieces of cotyledons and hypocotyls of one-week-old light-grown QAL seedlings and the *A. tumefaciens* strain GV3101. After co-cultivation, vancomycin (200 mg/l) was used instead of claforan to arrest bacterial growth. For selection of transgenic calli, hygromycin (10 mg/ml) was used instead of geneticin used in the original protocol.

### Generation of Arabidopsis roots

Roots of Arabidopsis wild type and *AtPSY* overexpressing lines were generated as described [58]. Seed were sterilized, sown on MS medium ([59]; MS salts, Gamborg's B5 vitamins, 1% [w/v] sucrose, and 0.25% [w/v] Phytagel), stratified for 4 days and grown for 7 days at 26°C under long-day conditions. Seedlings were transferred to B5 liquid medium (Gamborg's B5 salts, Gamborg's B5 vitamins, and 2% [w/v] glucose) and incubated for 14 days with shaking at 120 rpm under long-day conditions at 26°C. Plantlets were removed from the liquid, green plant parts were carefully removed and roots immediately ground in liquid nitrogen and stored at −70°C for further analysis.

### Protoplast generation

Protoplasts from Arabidopsis SDC and roots were generated as described [60]. Light microscopy was performed using an Axioskop 2 microscope (Carl Zeiss, Jena, Germany) equipped with the Axiovision 4.6.3 software.

### TaqMan Real-Time RT-PCR Assay

Total RNA was isolated using the plant RNA purification reagent (Invitrogen, Heidelberg, Germany). RNA purification, DNase I digestion and Real-Time RT-PCR assays were performed as described [19]. Primers and 6FAM-labeled probes were designed using Primer Express software (Applied Biosystems, Darmstadt, Germany) and are given in Table S1.

### Carotenoid Extraction and Quantification

Carotenoids were extracted using lyophilized plant materials and analyzed by HPLC as described [19]. The following approximate dry weights were used: 30 mg SDC; 5 mg seedlings; 70 mg Arabidopsis roots; 100 mg white carrot roots; 20 mg orange carrot roots.

### Generation of anti-AtPSY antibodies

*AtPSY* cDNA was amplified without the transit peptide coding sequence by PCR using mutagenized primers (position 574 according to accession no NM_121729). The PCR product was subcloned into the vector *pCOLDI* (Takara-Clontech, Heidelberg, Germany) to yield an N-terminal 6*His tag fusion. 6*His-AtPSYdTP inclusion bodies were isolated as described [19], dissolved in 10 ml 6 M Gu-HCl, 0.1 M Na_2_HPO_4_ (pH 7.15), the protein purified using HIS-Select Spin Columns (Sigma, Taufkirchen, Germany) and dialyzed against water. The protein precipitate was dissolved in SDS sample buffer, subjected to SDS-PAGE and gel slices containing 6*His-AtPSYdTP were used to immunize rabbits.

For the affinity purification of antibodies 1 ml of Affi-Gel 10 gel (Bio-Rad, München, Germany) was incubated overnight with 2 ml of 10 mg/ml 6*His-AtPSYdTP in coupling buffer (4 M Gu-HCl, 20 mM HEPES-KOH, pH 7.0). Blocking was achieved with 1 M ethanolamine-HCl, pH 8.0 for 4 h followed by sequential washing steps with 5 ml each of: TBS, buffer 1 (20 mM Tris-HCl pH 7.5, 500 mM NaCl), buffer 2 (50 mM glycine-HCl pH 2.3, 500 mM NaCl), buffer 3 (50 mM Tris-HCl, pH 8.8), two times TBS. For antibody binding, 5 ml of serum, diluted 1:10 in TBS, was passed three times over the column. The column was washed twice with 5 ml of TBS and buffer 1. Antibodies were eluted 10 times with 1 mL buffer 2 into reaction tubes containing 52 µl 1 M Tris-HCl, pH 9.0 and 5 µl 10 mg/ml BSA. Eluates were stored at 4°C.

### Protein extraction and Western blot analysis

Proteins were extracted with phenol as described [61]. Protein concentration was determined with Bio-Rad protein assay. After SDS-PAGE, blotting onto PVDF membranes (Carl Roth, Karlsruhe, Germany) and treatment with blocking solution (TBS containing 5% [w/v] milk powder), membranes were incubated with affinity-purified anti-AtPSY antibodies in PBS containing 0.1% (v/v) Tween-20 and 1% (w/v) milk powder for 2 h. For detection, the ECL system (GE Healthcare, München, Germany) was used. Western blots were stripped and reprobed with anti-actin antibodies (Sigma). Signal quantification was done using the Quantity One software (Bio-Rad).

## Supporting Information

Figure S1 Expression levels of carotenogenic enzymes in *35S::AtPSY* lines. Expression levels of carotenogenic enzymes were determined by Real-Time RT-PCR using total RNA isolated from roots of wild type and *35S::AtPSY* lines#12 and #22. Transcript levels were normalized to 18S rRNA level of the corresponding sample and expressed relative to the content in the wild type. *PDS*, phytoene desaturase, *ZDS*, ζ-carotene desaturase, *eLCY*, lycopene ε-cyclase; *bLCY*, lycopene β-cyclase; *bHYD1/2*, β-carotene hydroxylase 1/2.
(0.22 MB TIF)

Figure S2 Crystal formation in roots of *AtPSY*-overexpressing lines. Light microscopy (A) and polarization microscopy (B) images of a root protoplast prepared from *35S::AtPSY* line#12.
(1.28 MB TIF)

Table S1 Primers and probes used for Real-Time RT-PCR. “MGB probe” indicates the use of a Taqman minor groove binding probe, while Taqman probes are indicated with “probe”. Arabidopsis Genome Initiative (AGI) numbers and GenBank accession numbers (Daucus) are given in brackets below the gene names.
(0.05 MB DOC)
