# Supplementary figures and images for: Carotenoid Crystal Formation in Arabidopsis and Carrot Roots Caused by Increased Phytoene Synthase Protein Levels
Source: PLoS One. 2009 Jul 28;4(7):e6373. doi: 10.1371/journal.pone.0006373 (PMC2712097; doi:10.1371/journal.pone.0006373)

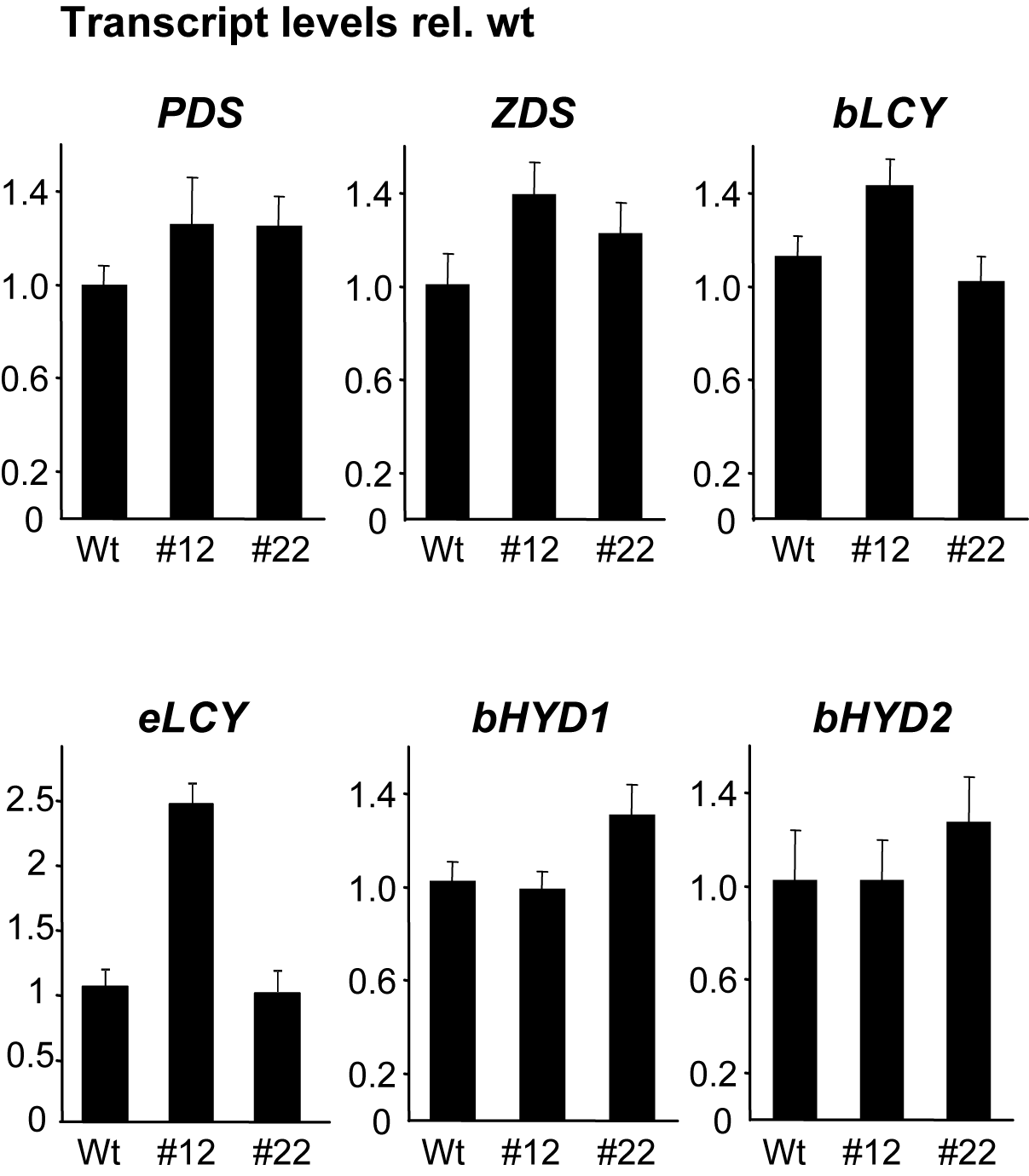

Supplement: Figure S1 — Expression levels of carotenogenic enzymes in 35S::AtPSY lines. Expression levels of carotenogenic enzymes were determined by Real-Time RT-PCR using total RNA isolated from roots of wild type and 35S::AtPSY lines#12 and #22. Transcript levels were normalized to 18S rRNA level of the corresponding sample and expressed relative to the content in the wild type. PDS, phytoene desaturase, ZDS, ζ-carotene desaturase, eLCY, lycopene ε-cyclase; bLCY, lycopene β-cyclase; bHYD1/2, β-carotene hydroxylase 1/2. (0.22 MB TIF) [file pone.0006373.s001.tif]

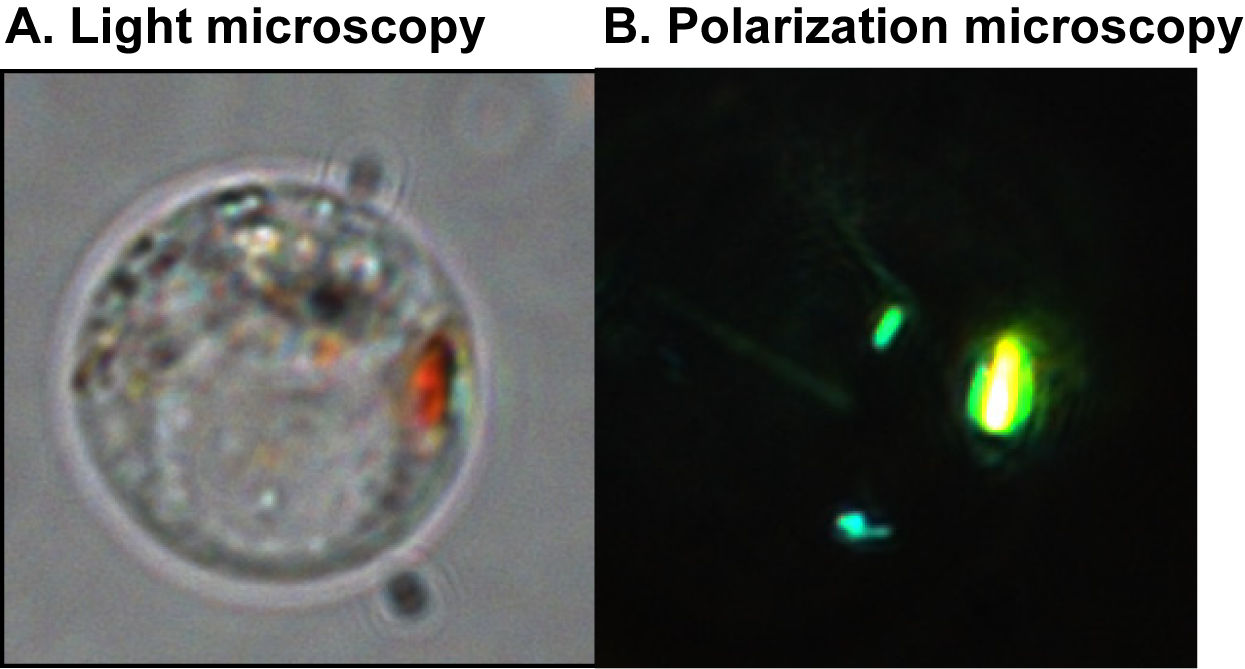

Supplement: Figure S2 — Crystal formation in roots of AtPSY-overexpressing lines. Light microscopy (A) and polarization microscopy (B) images of a root protoplast prepared from 35S::AtPSY line#12. (1.28 MB TIF) [file pone.0006373.s002.tif]
