# Supplementary material for: Carotenoid Crystal Formation in Arabidopsis and Carrot Roots Caused by Increased Phytoene Synthase Protein Levels
Source: PLoS One. 2009 Jul 28;4(7):e6373. doi: 10.1371/journal.pone.0006373 (PMC2712097; doi:10.1371/journal.pone.0006373)
Supplement: Table S1 — Primers and probes used for Real-Time RT-PCR. “MGB probe” indicates the use of a Taqman minor groove binding probe, while Taqman probes are indicated with “probe”. Arabidopsis Genome Initiative (AGI) numbers and GenBank accession numbers (Daucus) are given in brackets below the gene names. (0.05 MB DOC) [file pone.0006373.s003.doc]

| Gene Name | Primer/Probe | Sequence (5’ to 3’) |
| --- | --- | --- |
| *AtPSY* | forward | GCTGGAACCGTCGGATTG |
| **(At5g17230)** | reverse | TCGGTTGTTGCTTTCGACTTAG |
|  | probe | CGTTCCGGTTATGGGAATCGA |
| *AtPDS* | forward | GTTGCACTTCCCCACCTAGTG |
| **(At4g14210)** | reverse | CCTCCGGAAAGGCTTTGTATG |
|  | MGB probe | TCGAATATGATCCACTACTG |
| *AtZDS* | forward | AGATCCATTCAGACCCGATCAG |
| **(At3g04870)** | reverse | TGAACCGGCGAGGAAGAA |
|  | MGB probe | AGACGCCCATAAAGA |
| *AteLCY* | forward | CCAACTTCGTATTGCAAGAGCTT |
| **(At5g57030)** | reverse | CTGCAGGCTTGCCACTGTT |
|  | MGB probe | CCCGAAGCTGCTCC |
| *AtbLCY* | forward | CCTCTGTCTACGCCGTTCGA |
| **(At3g10230)** | reverse | ATCAACTCTCTGCTGAGGTTTGG |
|  | MGB probe | AGGCCACAAGTCTC |
| *AtbHYD1* | forward | CTCGTGCACAAGCGTTTCC |
| **(At4g25700)** | reverse | GGCGACCTTTCGGAGGTAAG |
|  | MGB probe | TGTAGGTCCCATCGCCGA |
| *AtbHYD2* | forward | TTCTCCGCAAACCACCCTATA |
| **(At5g52570)** | reverse | AGCCGTTGAATCTTAGAGATGGA |
|  | MGB probe | CCACCGCAGTTTT |
| *DcPSY1* | forward | GAGCTAGTAGACGGGCCTAATGC |
| **(DQ192186)** | reverse | TTCAGCCTCTTCTCCCATCTG |
|  | probe | TCCCATATCACGCCCAAGGCTCTTG |
| *DcPSY2* | forward | ACTCCTATTTGTGCTTCATCGAAA |
| **(DQ192187)** | reverse | GAAGGTTACCGATAAATGGAGGAAT |
|  | probe | ACATCCTTGCCCTCTTAATTTGCTTCTTCATGA |
